# Action of disinfectant solutions on adaptive capacity and virulence factors of the Candida spp. biofilms formed on acrylic resin

**DOI:** 10.1590/1678-7757-2021-0024

**Published:** 2021-09-03

**Authors:** Mauricio Malheiros BADARÓ, Frank Lucarini BUENO, Lais Ranieri MAKRAKIS, Camila Borba ARAÚJO, Viviane de Cássia OLIVEIRA, Ana Paula MACEDO, Helena de Freitas Oliveira PARANHOS, Evandro WATANABE, Cláudia Helena SILVA-LOVATO

**Affiliations:** 1 Universidade Federal de Santa Catarina Departamento de Odontologia FlorianópolisSC Brasil Universidade Federal de Santa Catarina (UFSC), Departamento de Odontologia, Florianópolis, SC, Brasil.; 2 Universidade José do Rosário Vellano Departamento de Odontologia AlfenasMG Brasil Universidade José do Rosário Vellano (UNIFENAS), Departamento de Odontologia, Alfenas, MG, Brasil.; 3 Universidade de São Paulo Faculdade de Odontologia de Ribeirão Preto Departamento de Materiais Dentários e Prótese Ribeirão PretoSP Brasil Universidade de São Paulo (USP), Faculdade de Odontologia de Ribeirão Preto, Departamento de Materiais Dentários e Prótese, Ribeirão Preto, SP, Brasil.; 4 Universidade de São Paulo Faculdade de Odontologia de Ribeirão Preto Departamento de Odontologia Restauradora Ribeirão PretoSP Brasil Universidade de São Paulo (USP), Faculdade de Odontologia de Ribeirão Preto, Departamento de Odontologia Restauradora, Ribeirão Preto, SP, Brasil.

**Keywords:** Candida, Acrylic resins, Biofilms, Microbial viability, Denture cleansers

## Abstract

Understanding the behavior of *Candida* spp. when exposed to denture disinfectants is essential to optimize their effectiveness. Changes in the virulence factors may cause increased resistance of *Candida* spp. to disinfectant agents.

**Objective:**

To evaluate the microbial load, cellular metabolism, hydrolytic enzyme production, hyphae formation, live cell and biofilm quantification of *Candida albicans, Candida tropicalis* and *Candida glabrata* after exposure to disinfectant solutions.

**Methodology:**

Simple biofilms were grown on heat-polymerized acrylic resin specimens, and divided into groups according to solutions/strains: distilled water (control); 0.25% sodium hypochlorite (NaOCl 0.25% ); 10% *Ricinus communis* (RC 10%); and 0.5% Chloramine T (CT 0.5%). The virulence factors were evaluated using the CFU count (microbial load), XTT method (cell metabolism), epifluorescence microscopy (biofilm removal and live or dead cells adhered), protease and phospholipase production and hyphae formation. Data were analyzed (α=0.05) by one-way ANOVA/ Tukey post hoc test, Kruskal-Wallis test and Wilcoxon test.

**Results:**

NaOCl 0.25% was the most effective solution. CT 0.5% reduced the number of CFUs more than RC 10% and the control. RC 10% was effective only against *C. glabrata*. RC 10% and CT 0.5% decreased the cellular metabolism of *C. albicans* and *C. glabrata*. Enzyme production was not affected. Hyphal growth in the RC 10% and CT 0.5% groups was similar to that of the control. CT 0.5% was better than RC 10% against *C. albicans* and *C. tropicalis* when measuring the total amount of biofilm and number of living cells. For *C. glabrata*, CT 0.5% was equal to RC 10% in the maintenance of living cells; RC 10% was superior for biofilm removal.

**Conclusions:**

The CT 0.5% achieved better results than those of *Ricinus communis* at 10%, favoring the creation of specific products for dentures. Adjustments in the formulations of RC 10% are necessary due to efficacy against *C. glabrata*. The NaOCl 0.25% is the most effective and could be suitable for use as a positive control.

## Introduction

The complete denture is made with acrylic resin and widely used to replace all teeth, restoring function, aesthetics and comfort to the patient. However, acrylic resin in the oral environment favors the adherence of oral debris, bacteria, and *fungi*. Thus, there is a direct risk relationship between denture use, biofilm and the development of denture stomatitis (DS).^1^ Many individuals are unaware that they have DS, an infection that can aggravate systemic diseases, such as chronic obstructive pulmonary disease, bacterial endocarditis, aspiration pneumonia and gastro-intestinal infection, due the high *Candida* spp. count.^2,3,4^ Immune response is a determinant of infections caused by *Candida* spp., which can become even more serious with the exacerbation of the pathogenicity of these microorganisms and imbalance of the individual’s immune response.^3^*Candida* infections are predominantly caused by *C. albicans*, but the literature indicates an increasing role for *non-albicans Candida* species (*C. glabrata, C. tropicalis, C. parapsilosis* or *C. krusei*) as infectious agents, which have different adaptive capacity, different susceptibility to antifungal agents^3,5^ and potential for adhesion on acrylic surfaces.^6,7^ Therefore, the relative value of cell surface hydrophobicity and the biofilm biomass of *non-albicans Candida* species is greater than *C. albicans*, considering that 92% of the *non-albicans Candida* species of oral isolates had the capacity to form biofilm against only 78% of *C. albicans*.^8^ This fact indicates the need for a comprehensive approach to *non-albicans* species in individuals wearing dentures.

The adaptive capacity of *Candida* is influenced by virulence factors, such as the adhesion, biofilm formation, microbial load and cell viability, cellular metabolism, hydrolytic enzymes production, and phenotypic changes with formation of hyphae.^9,10^Change in the adaptive capacity is evident when infection is recurrent after therapy with antimicrobial agents, indicating resistance of the microorganism^1^ and limiting treatment options. Infection control is even more difficult for denture users with inadequate hygiene and the presence of biofilm is clinically significant. Thus, disinfectant solutions have been proposed for the prevention of DS and for microbiota control^1,12,13^ to reduce the need for systemic antifungal use.

Sodium hypochlorite (NaOCl) has proven antimicrobial activity^1,13-17^ and has been recommended as a disinfecting agent for dentures by the American Dental Association (ADA)^18^. Satisfactory results have been reported with NaOCl at 0.25% concentration, which maintained the antimicrobial action, the ability to remove the biofilm^1,13,16,17^ and prevent damage to the acrylic resin.^19^ However, NaOCl may cause allergic reactions,^20^ oxidation of metals, and clothing staining. Alternative disinfectants such as *Ricinus communis*^1,13,16,19,21^ and Chloramine T^1,22-25^ have been proposed as alternative solutions.

The detergent derived from *R. communis* oil causes loss of cytoplasmic constituents and fungal cell death, damaging the fungal cell wall.^26^ The use of *R. communis* alongside brushing promoted the remission of DS,^1,12,13^ in which the concentration of 10% of *R. communis* was better than NaOCl at 0.25%.^13^ Moreover, a solution of *R. communis* in different concentrations has been used to remove biofilm from complete denture.^1,13,21^ Chloramine T promotes oxidative reactions, enables the hydrolysis of proteins, and destroys the cell material from microorganisms.^27^ It is a common component of oral antiseptics, and, when incorporated into dentifrices is effective at removing denture biofilm;^22-24^ the Chloramine T solution showed efficacy in biofilm removal of the titanium specimens.^25^ However, only one study has evaluated this solution as an immersion agent for the daily hygiene of complete dentures, with promising findings for 0.5% concentration.^1^

Although there are clinical studies with these solutions,^1,13,16^ this laboratory study is important to prove that the use of these disinfectant solutions does not cause adaptation or resistance of the microorganisms. The originality is the absence of researches verifying the adaptive capacity of *Candida* spp. regarding virulence and biofilm factors after contact with disinfectants in concentrations with clinical feasibility. Therefore, given the need to establish effective preventive measures against *Candida* spp., this study aimed to evaluate the influence of NaOCl at 0.25%, *R. communis* at 10% and Chloramine T at 0.5% solutions on the microbial load, cellular metabolism, hydrolytic enzyme production, hyphae formation, live cell quantification and biofilm removal of *Candida* spp. on acrylic resin as alternative disinfectants for dentures against *Candida albicans, Candida tropicalis* and *Candida glabrata.* The study hypothesis was that the solutions would not differ significantly from each other; however, they would differ from the negative control.

## Methodology

### Specimen preparation and experimental design

Heat-polymerized acrylic resin (Artigos Odontológicos Clássico Ltda., São Paulo, SP, Brazil) was mixed, packed in circular molds (13×3 mm) and conventionally polymerized.^17^ The flask was placed in room temperature water, which reached 65°C in 1 hour. Thereafter, the temperature was raised to 100°C in half an hour and held for 1 hour, and then lowered to room temperature. The specimens were finished using rotary instruments and 180-grit abrasive paper in a polishing machine (Arotec, Aropol E, Cotia, SP, Brazil).

To simulate the inner surface of the denture, the surface roughness (Ra) was standardized between 2.7 and 3.7 µm^28^ with a profilometer (Surftest SJ-201P, Mitutoyo Corporation, Kawasaki, Japan). Three readings were made, one in the central portion and two with 2 mm to the right and left (5 “cutoff” of 0.8 μm) of the center. The specimens were randomly divided into 12 groups (n=24) according to strains frequently isolated in clinical studies.^1,16^ Reference strains were used, which are: *C. albicans* (ATCC 10231), *C. tropicalis* (ATCC 750) and *C. glabrata* (ATCC 2001); and solution [C: distilled water - Negative control; NaOCl 0.25% : 0.25% sodium hypochlorite (Injectcenter, Ribeirão Preto, SP, Brazil); RC 10%: 10% *R. communis* (Institute of Chemistry of São Carlos, University of São Paulo, SP, Brazil); CT 0.5%: 0.5% Chloramine T (Lioserum Chemicals, Ribeirão Preto, SP, Brazil)].^1^ The *R. communis* solution was made by an esterification reaction, consisting of triglycerides containing fatty acids: ricinoleic acid; linoleic acid; stearic acid; palmitic acid; dihydroxystearic acid; licosanoic acid; linolenic acid.^22^ CT 0.5% solution (tosylchloramide-sodium; molecular weight: 281.69 g/L; reagent grade; Merck) was prepared according to Arnitz, Nagl, Gottardi^29^ (2009) by dissolving it in phosphate-buffered saline - PBS (pH 7.1).

In each group, nine specimens were used to assess microbial load (CFU), enzymatic activity and hyphae formation; nine to assess metabolic activity (XTT); and three for fluorescence microscopy. To confirm the sterility of the procedures and materials, three sterilized specimens were put in sterilized culture medium, and, to confirm biofilm formation, another three specimens contaminated by *Candida* spp. and not immersed in disinfection solution were used. Before biofilm formation, the specimens were sterilized by irradiation in a microwave oven (650W; 6 minutes).^30^ The experiments were performed in triplicate on three different occasions.

### Mono-species biofilm formation

*Candida* strains were reactivated in Sabouraud Dextrose Agar (HiMedia Laboratories Pvt. Ltda., Mumbai, India) at 37°C (48 hours), transferred to Sabouraud Dextrose Broth (HiMedia Laboratories Pvt. Ltda, Mumbai, India) and incubated (37°C/18 hours). An aliquot of the culture medium with the reactivated strains was diluted in PBS (100× dilution; 990 µL PBS plus 10 µL of the microorganism suspension), from which 10 µL were transferred to the Neubauer chamber (HBG). The standardization in 1×10^6^ CFU/mL of strains was done after counting the outer quadrants in the Neubauer chamber under a microscope, in which the average of the counts is multiplied by the dilution and converted to mL. In a laminar flow chamber, the specimens were placed in 24-well culture plates with 1.5 mL of the inoculated culture medium and incubated (37ºC; 90 minutes; 75 rpm). The specimens and wells were washed with phosphate buffered saline (PBS) to remove the non-adherent microorganisms, filled with sterile culture medium (1.5 mL) and incubated for the formation and growth of biofilm at 37°C for 48 hours under 75 rpm agitation. After the first 24 hours of incubation, half of the culture medium was replaced with the same amount of sterile medium (to avoid saturation), which remained for another 24 hours to complete the biofilm formation cycle.

### Evaluation of microbial load

After the mono-species biofilms growth, the specimens were immersed in the disinfectant solutions (20 minutes),^1,12,13,16,17^ rinsed in PBS (three times) and transferred to test tubes containing Letheen medium (Difco Laboratories Inc., Detroit, Michigan, USA). The tubes were placed in an ultrasonic vessel (20 minutes; 200 Watts – Altsonic, Clean 9CA, Ribeirão Preto, SP, Brazil) and Vortex agitator (1 minute – Phoenix^®^ - AP56, Ind. and Com., Araraquara, SP, Brazil). Serial dilutions (10^0^ to 10-^3^) were seeded in Sabouraud Dextrose Agar and incubated (37°C; 48 hours). The following formula was used to calculate the microbial load: CFU/mL=number of colonies×10^n^/q, where n is the absolute value of dilution (0, 1, 2, or 3) and q is the quantity (mL) pipetted for each dilution at inoculation (0.05 mL). After 24 hours of incubation, the aliquots were used in hydrolytic enzyme and hyphae formation assays.

### Evaluation of cell metabolism

The cell metabolism was evaluated by XTT assay [2,3-bis (2-methoxy-4-nitro-5-sulfophenyl)-2H-tetrazolium-5-carboxanilide]. The specimens with biofilm growth were subjected to disinfection procedures and transferred to sterile 24-well culture plates containing a solution composed of PBS supplemented with 100 mM glucose (948 μL; Sigma Aldrich, Sigma-Aldrich Corporation, St Louis, Missouri, USA), XTT solution and menadione (252 μL; Sigma Aldrich, Sigma-Aldrich Corporation, St Louis, Missouri, USA).^31^ The plates were protected from light during incubation (37°C, 2 h). Subsequently, a 100-μL aliquot was transferred from each well to 96-well plates, in triplicate, for three readings in a microplate reader (Multiskan GO, Thermo Fisher Scientific, Waltham, Massachusetts, USA) at 492 μm.^31^

### Evaluation of enzyme production and hyphae formation

After the biofilm had formed for 48 hours, the specimens were exposed to disinfectant solutions and transferred to the Letheen culture medium, and the tube was incubated for 24 hours. Next, the cellular concentration was counted in a Neubauer chamber. The cell suspension (1 mL) was centrifuged (6000 rpm, 5 minutes). The supernatant was destined for proteinase analysis and the pellet, for phospholipase analysis. For proteinase quantification the EnzChek^®^ Protease Assay fluorimetric kit (Molecular Probe E6638, Eugene, Oregon, USA) was used according to the manufacturer’s instructions. Fluorescence was assessed with a microplate reader (Synergy II, BioTek Instruments, Winooski, VT, USA; excitation: 485 μm; emission: 538 μm). The values obtained were used in linear equations derived from standard curves, with the enzymatic activity in μL/mL. For phospholipase analysis, the fluorimetric kit Amplex^®^ Red Phosphatidylcholine-Specific Phospholipase C Assay (Molecular Probe, Eugene, Oregon, USA) was used according to the manufacturer’s instructions to transfer the working solution and cell lysate to a 96-well plate, in triplicate, which was protected from light, incubated (37°C; 3 hours), and then read in a microplate reader (excitation: 544 μm; emission: 590 μm). The values provided were compared with the fluorescence values of the positive controls provided by the manufacturer.

To assess hyphae formation, 3×10^6^ cells/ mL were transferred into 5 mL of hyphal-growth-inducing medium (medium 199; LGC Biotechnology, Cotia, SP, Brazil) containing 10% of the fetal bovine serum (LGC Biotechnology, Cotia, SP, Brazil) and incubated at 37°C under agitation of 80 rpm for 3 hours. A cell count (yeast + hyphae) was performed in a Neubauer chamber under an optical microscope (10× to 40× objective – Bio-Focus Saintifik, Petaling Jaya, Malásia). The formula used was hyphae/ mL = S/4 × D × 10^4^, where S is the sum of the major quadrants (Neubauer chamber); D is the dilution used for counting (10×); 10^4^ is the correction factor for unit (mL).

### Quantification of total biofilm and living cells

Epifluorescence microcopy was used to assess the presence of viable and dead cells adhered on the surface of the specimens. After disinfection, specimens (n=3, per group/ strain) were transferred to a 24-well plate containing 1.2 mL of Live/Dead^®^ dye (Live/Dead^®^ BacLight™ kit – Life Technologies of Brazil Com. Ind. Prod. Biotec. Ltda., Itapevi, SP, Brazil).^31^ The plate was incubated at room temperature for 15 minutes under light protection. The specimens were placed on individual coverslips for analysis under an inverted fluorescence microscope with appropriate filters: FS38HE (green staining); FS43HE (red staining) and a 63× objective (inverted microscope Observer A1 – Carl Zeiss, Oberkochen, Germany). The specimen manipulation, sterile and individual forceps were used only contacting the sides and avoiding the analysis surface. The different cell stains were used to quantify the live (stained in green; Syto 9) and dead (stained red; Iodide) microorganisms, and the sum of the two to quantify the total biofilm present on the surface of the specimen. Twenty random images of the surface of each specimen were captured under a microscope (63× magnification; software Zen Lite 2.3 – Carl Zeiss, Oberkochen, Germany). The AxioVision software program (AxioVision release 4.8.2 – Carl Zeiss, Oberkochen, Germany) was used to measure the total image area (13733.82 mm^2^) and the stained areas. The calculation of live cells’ area considered the difference between the green and the red (dead cells) pigmented area.^31^ Then, the following formula was applied for conversion to percentage: Percentage of live cells = Live cells’ area/Total image area × 100; for the calculation of the total biofilm area, only the value of the area of the cells stained green was used. Total biofilm percentage = Biofilm area/Total image area × 100.^31^

### Data analysis

After verifying a normal (Shapiro-Wilk test) and homogeneous (Levene test) distribution, the data were analyzed (α=0.05) as follows: ANOVA (one-way) and the Tukey post hoc test for microbial load; the Kruskal-Wallis test and stepwise step-down post hoc test for cell metabolism; the Kruskal-Wallis test for enzyme production and hyphae formation; and the Kruskal-Wallis and Wilcoxon test for comparison of the solutions and the living biofilm, and the total biofilm, respectively; descriptive analyses were used for the fluorescence images. SPSS 21.0 (SPSS Inc.) was used considering a 95% confidence interval.

## Results

### Microbial load and cell metabolism

**Figure 1:**
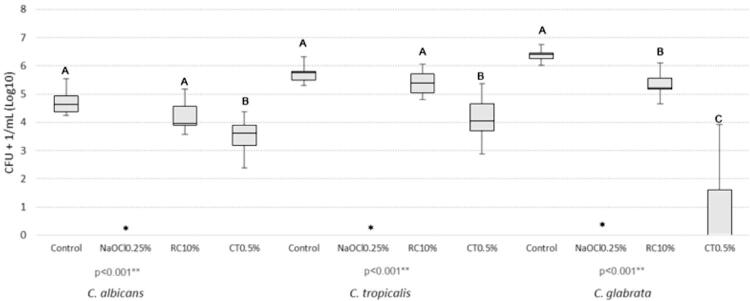
Comparative analysis of microbial load (CFU + 1/ mL, log10) of *Candida* spp. after immersion of the specimens in the disinfectant and control solutions

**Figure 2:**
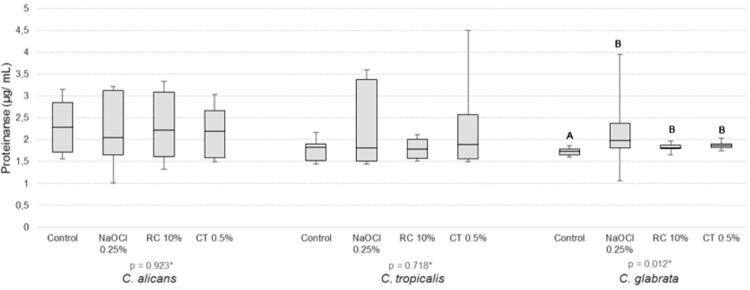
Comparative analysis of metabolic activity (absorbance 492 nm) of *Candida* spp. after immersion of the specimens in the disinfectant and control solutions

The NaOCl 0.25% reduced to zero the CFU count and the cellular metabolism of all *Candida* species. CT0.5% was more effective in decreasing the CFU count when compared with RC 10% and the negative control. RC 10% differed from the distilled water (negative control) only against *C. glabrata* (Figure 1). RC 10% and CT 0.5% decreased the cellular metabolism of *C. albicans* and *C. glabrata* in a similar manner, but, for *C. tropicalis*, CT 0.5% was more effective (Figure 2).

* Sodium hypochlorite (NaOCl 0.25%) reduced the number of CFU to zero; ** Anova (one-way); post-test of Tukey; ^AB^ Equal capital letters indicate statistical similarity between groups.

* Sodium hypochlorite (NaOCl 0.25%) reduced the number of metabolic activity to zero; ** Kruskal-Wallis, stepwise step-down post-test; ^AB^ Equal capital letters indicate statistical similarity between groups.

### Enzyme production and hyphae formation

**Figure 3:**
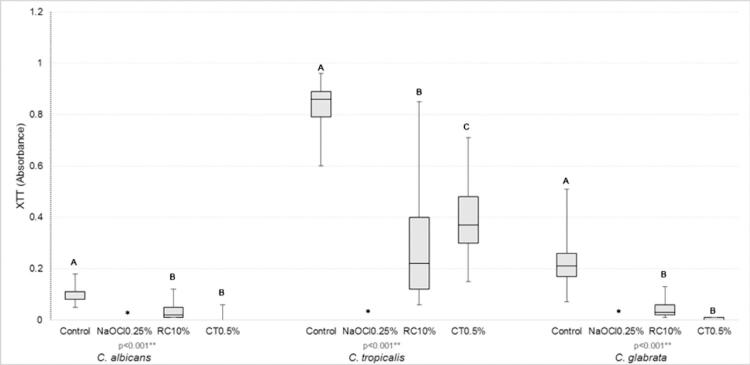
Comparison of proteinase (µg/ mL) production by *Candida* spp. after immersion of the specimens in the disinfectant solutions and water (Control)

**Figure 4:**
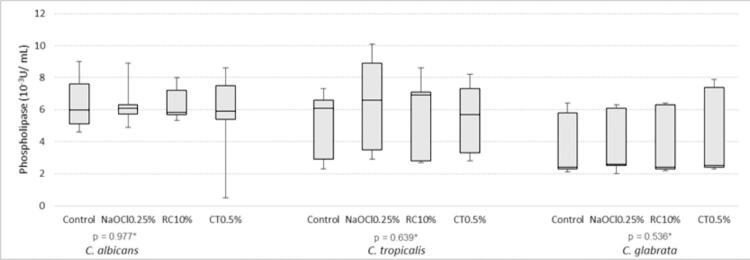
Comparison of phospholipase (10-3U/ mL) production by *Candida* spp. after immersion of the specimens in the disinfectant solutions and water (Control)

**Figure 5:**
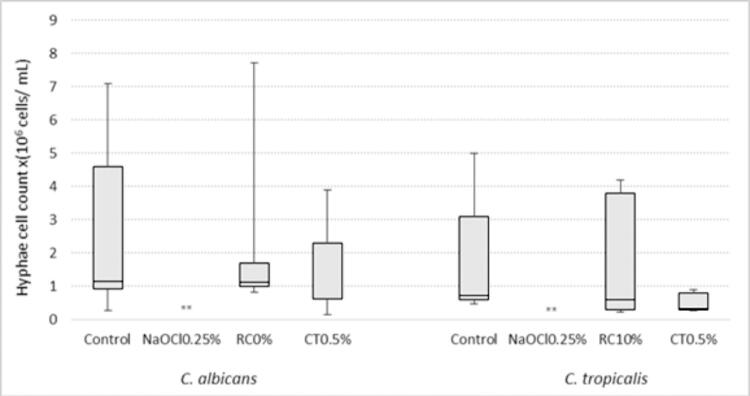
Comparison hyphae cell count (x106 cells/ mL) of *Candida* spp. after immersion of the specimens in the disinfectant solutions and water (Control)

Only *C. glabrata* showed increased proteinase production after immersion in all solutions tested when compared with negative control (Figure 3). No difference was found in phospholipase production by *Candida* spp. after immersion of the specimens in the different disinfectant solutions (Figure 4). The NaOCl 0.25% reduced hyphal growth to zero. Hyphal growth in the other groups of solutions was similar to that in the control (Figure 5). Even though they were statistically similar, the values of CT0.5% were lower than those of the RC10%.

*Kruskal-Wallis test and post-test stepwise step-down; ^AB^ Equal capital letters indicate statistical similarity between groups.

*Kruskal-Wallis test.

*Kruskal-Wallis test; ** 0.25% sodium hypochlorite reduced the number of cells in the form of hyphae to zero.

### Quantification of total biofilm and living cells adhered

NaOCl 0.25% showed the best results. CT 0.5% was better than RC 10% against *C. albicans* and C. tropicalis for the total amount of biofilm and number of living cells. For *C. glabrata*, CT 0.5% was equal to RC 10% in the maintenance of living cells, where as RC 10% was better for biofilm removal (Table 1).

**Table 1:** Comparison (mean and SD, Median and CI) of the living biofilm area and total biofilm area, in percentage, after immersion of the specimens in the disinfectant and control solutions

Groups	*C. albicans*			*C. tropicalis*			*C. glabrata*		
	Living biofilm	Total biofilm	p**	Living biofilm	Total biofilm	p**	Living biofilm	Total biofilm	p**
C	50.31±19.85	51.31±19.34	0.001	13.65±4.06	14.98±3.7	0.000	16.07±9.22	38.36±8.12	0.00
	57.5 (41.0;59.6)^Aa^	57.72 (42.3;60.4)^Ab^		14.0 (11.7;15.5)^Aa^	15.4 (13.2;16.7)^Ab^		18.3 (11.8;20.4)^Aa^	36.1 (34.6;42.2)^Ab^	
NaOCl 0.25%	0 (-;-) †^a^	3.83±3.25	0.000	0(-;-)†^a^	1.28±1.21	0.000	0(-;-)†^a^	0.14±0.21	0.008
		3.23 (2.3;5.4)^Db^			1.0 (0.7;1.8)^Cb^			0.0 (0.04;0.2)^Db^	
RC 10%	10.31±6.64	11.58±7.66	0.000	6.67±2.02	8.26±1.85	0.000	0.96±1.21	3.97±1.09	0.000
	9.7 (7.2;13.4)^Ba^	10.4 (8.0;15.2)^Bb^		6.8 (5.7;7.6)^Ba^	7.9 (7.4;9.1)^Bb^		0.32 (0.4;1.5)^Ba^	4.07 (3.5;4.5)^Cb^	
CT 0.5%	0.44±0.67	7.34±6.23	0.000	0.03±0.1	1.56±0.9	0.000	2.6±3.19	20.91±10.15	0.000
	0.0 (0.12;0.7)^Ca^	5.7 (4.4;10.2)^Cb^		0.0 (-0.01;0.08)^Ca^	1.6 (1.1;2.0)^Cb^		0.2 (1.1;4.1)^Ba^	19.45 (16.2;25.7)^Bb^	
p*	<0.001	<0.001		<0.001	<0.001		<0.001	<0.001	

† the sanitizer reduced the biofilm to zero. * Kruskal-Wallis test. AB Equal capital letters indicate statistical similarity between lines; ** Wilcoxon test. ab Equal lowercase letters indicate statistical similarity between columns.

**Figure 6:**
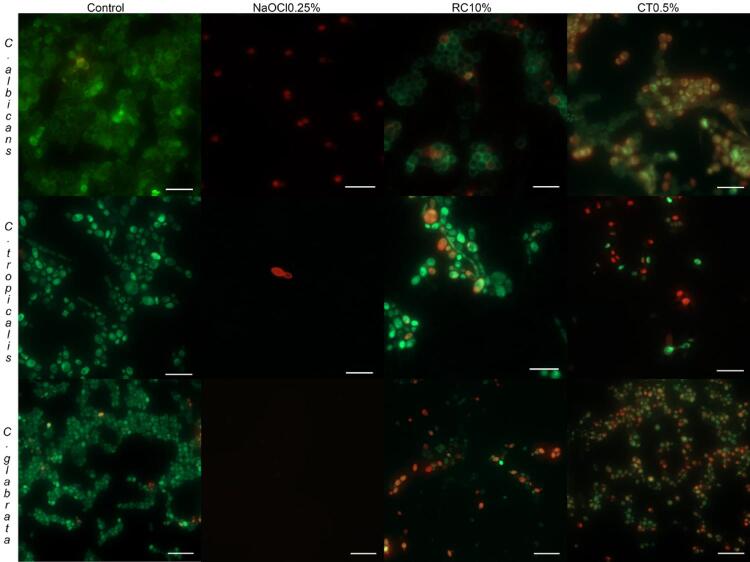
Epifluorescence microscopy images of the *C. albicans, C. tropicalis* and *C. glabrata* biofilm in the specimens after immersion in disinfectant and negative control (Scale bar = 20µm)

Qualitative analyses of the epifluorescence microscopy images indicated that NaOCl 0.25% removed almost 100% of the cells from the different *Candida* species, and the cells that remained adhered were dead. For the control group, a large quantity of living cell biofilm was seen covering the specimen. The images of the specimens after immersion in *Ricinus communis* and Chloramine T demonstrated the presence of biofilm with live cells (green color) and dead cells (red color), indicating the intermediate action of these solutions (Figure 6).

## Discussion

In this study, ATCC strains were used to reduce the great diversification of factors influencing the analyzed variables, such as the expressive variability among organisms (hosts/ human physiology). As well as between clinical strains of the same species of *Candida*, which can show differences in the cell surface hydrophobicity and adhesion,^7,32^ contributing in several states of disease in the human host, since superficial until and systemic infections.^6^ Thus, this study cannot provide a representation of the microorganisms found in the oral cavity, due to its non-clinical origin. However, it can be considered the first step towards an in-depth understanding of the mechanisms that permeate the relationship between *Candida* spp. and disinfectant agents for dentures. Future researches using mixed biofilms and strains with clinical origin can be proposed to provide the representativeness and clinical viability of the process of controlling *Candida* species in users of dentures.

The hypothesis was partially accepted. The NaOCl 0.25% presented the highest efficiency, and the experimental groups (RC 10% and CT 0.5%), in some situations, were similar to the negative control (distilled water). NaOCl was the most effective in this study, and the results were consistent with the literature because of its demonstrated *in vivo* and *in vitro* antimicrobial efficacy,^1,13,16,17^ adequate removal of biofilm, satisfaction of use by denture users,^13^ remission of denture stomatitis,^1,13^ and acceptable changes in the mechanical properties of the heat-polymerized acrylic resin.^19^ Therefore, the NaOCl at 0.25% could be considered the positive control or gold standard in other research.

*R. communis* was efficient only against *C. glabrata*, having a lesser effect than the other solutions. The literature shows different results with *R. communis*, with a study that corroborates the results found in this study^12^ and other that indicates promising results.^13,16,17^*R. communis* had a minimum inhibitory concentration of 0.0781% for *C. albicans* and *C. glabrata* and, when incorporated into a dentifrice, had a significant effect against bacteria (*S. mutans; B. subtilis*) that are important in biofilm formation.^22^ The contrasting results may come from differences in the susceptibility of the microorganisms when in contact with the disinfectants or from methodological differences. The effects of *R. communis* after long periods of immersion (overnight, 8 hours) could differ, and mixed biofilms could also lead to different results. Therefore, additional studies are necessary once *R. communis* is a natural products and it can be one therapeutic alternatives in the treatment of denture stomatitis^1,13^ with low-cost and lower adverse effects as compared to antifungal drugs. CT 0.5% was better than RC 10%, and the negative control in terms of decreasing microbial load. Verardi, et al.^25^(2016) also reported a reduction of CFU/mL with titanium specimens after using the Chloramine T solution, which destroys the cellular material or disrupts important cellular processes of the microorganisms by oxidative reactions.^27^ Microorganisms do not become resistant to Chloramine T as can happen with antibiotics and is effective even at low concentrations.^27^

The data on cellular metabolism confirmed the findings on microbial load. The NaOCl 0.25% had 100% effectiveness for both variables, and these results were in accordance with the literature. The solution of NaOCl at 1% (immersion time for 10 seconds) reduced the microbial load of *Candida* and decreased the cellular metabolism by 98% in a mixed biofilm model (*C. albicans, C. glabrata* and *S. mutans*)^28^, and caused 88% of the reduction in the *C. albicans* metabolism (simple biofilm) after 90 seconds of immersion.^15^ These findings confirm the feasibility of using NaOCl at 0.25% as a positive control with a 20-minute immersion time, since the efficacy was similar to that of higher concentrations. *R. communis* reduced the metabolism of *Candida* spp., but it was only significant enough to decrease the microbial load of *C. glabrata*. RC 10% and CT 0.5% had similar effects on the cellular metabolism of this specie but differed in terms of microbial load. Thus, only CT 0.5% presented coherent results regarding the reduction in cellular metabolism and microbial load. Studies using the same concentration of these solutions are scarce, limiting the available information about their mechanisms of action against *Candida albicans* and *non-albicans*.

The solutions did not change the hydrolytic enzyme production (proteinase and phospholipase) from *C. albicans* or *C. tropicalis*. One of the enzymatic functions is to allow the fungus to invade and cause damage to tissues.^10^ The maintenance of the production rates of these enzymes was similar to that of the negative control (distilled water) when *C. albicans* and *C. tropicalis* were exposed to hygiene solutions. This result can be considered good, since the solutions did not increase the pathogenicity of these species in the concentrations and immersion period: used in this study. However, *C. glabrata* showed an increase in proteinase production when exposed to all solutions. This result suggests a greater specific capacity of this species to react to a given stimulus.^33^ This characteristic can influence the survival or persistence of the microorganism between an initial exposure to an antimycotic agent and the acquisition of mutations that confer resistance, an adaptive response. *C. glabrata* has a complex population structure, with genomic variants that may arise during the process of adaptation to environmental changes and persist over time, giving this species a greater pathogenicity.^11^ Different results were reported by Marcos-Arias, et al.^34^ (2011), who stated that oral samples of *Candida* spp. showed low levels of proteinase production (<30%). According to Gümrü, et al.^35^(2006) only *C. albicans* showed changes in phospholipase production when compared with non-*albicans* species. Samaranayake, Raeside and Macfarlane^36^ (1984) indicated that *C. tropicalis* and *C. glabrata* did not produce detectable phospholipases, only *C. albicans* were phospholipase-positive.

The hyphae contribute to increasing the mass of *Candida* biofilm, and consequently increase the difficulty of removal^37^ and the capacity of invasion of the tissues.^10^ The hyphae formation and the production of proteinases are regulated in a coordinated way, since the cells of *C. albicans* in the form of hyphae require the support of hydrolytic enzymes to be totally invasive *in vivo*.^10^ Although the formation of hyphae does not occur with all *Candida* species, the synergism between the species guarantees adhesion and infection.^38^ Staniszewska, et al.^38^ (2012) reported that, in the blastoconidia form, the number of enzymes produced was reduced compared with situations in which the germ tube, pseudo-hyphae and true hyphae were observed. Thus, the non-significant alteration in proteinase production by *C. albicans* can be attributed to the lack of increase in hyphae after the contact with the disinfectant solutions in the present study. This was not the case for *C. glabrata*, which does not form hyphae, implying that this species has other means of producing proteinase, explaining the increase observed. The tests performed for these variables were carried out concomitantly with the same cellular content of each sample, reinforcing this hypothesis.

For biofilm removal and promotion of cell death, NaOCl 0.25% was the most effective solution. This finding is important since dead cells on the surface of the dentures act as an aggregation agent for the adhesion of new microorganisms and since the effective removal of biofilm is essential for the control of the inflammation promoted by *Candida*. This result is consistent with those of Silva, et al.^39^ (2011) and Gama, et al.^14^ (2015), who evaluated the presence of live cells and the ability to remove *C. albicans* biofilm after immersion in higher concentrations of NaOCl. The authors recommended an immersion time of 5 and 10 minutes, respectively, while the current study used an immersion time of 20 minutes, demonstrating that NaOCl can be effective at lower concentrations by remaining in contact with the biofilm for longer. The use of lower concentrations reduces the potential for damage to the dentures, especially the surface roughness, which would lead to higher adhesion of microorganisms. Badaró, et al.^19^ (2017) reported that the roughness of heat-polymerized acrylic resin remained clinically acceptable during immersion in NaOCl 0.25%.

Regarding biofilm removal, RC 10% reduced the amount of all species when compared with the negative control, but CT 0.5% was more effective against *C. albicans* and *C. tropicalis,* and less effective against *C. glabrata*. This may be because of the lack of hyphae in this species, which is thus more easily eliminated by the detergent action of *R. communis*. Sánchez-Vargas, et al.^40^ (2013) demonstrated that biofilm formation is dependent on *Candida* spp. and that, although *C. albicans* is more prevalent, biofilm production was higher in *C. glabrata* isolates, followed by *C. tropicalis, C. albicans* and *C. krusei*. These findings reinforce the importance of research such as the current study involving less prevalent species, but with a high potential for virulence. The present research evaluated study evaluated the effect of different solutions against *C. tropicalis.* The authors are unaware of previous studies that performed the same analysis. Limitations of the present study included the lack of evaluation using a mixed biofilm, clinical strains, mature biofilms and a long period of biofilm formation; this would be important for future studies and to facilitate the proximity to the clinical reality. Moreover, the results of this *in vitro* study are preliminary and important to show the behavior of the 3 *Candida* species evaluated when exposed to NaOCl, Chloramine T and *R. communis* disinfectants, but they must be interpreted with caution, since the general health of the host (immunological conditions) , local conditions of the oral cavity (quality and quantity of saliva; pH; hygiene), conditions of the dentures and individuals’ adherence to the proposed protocols, can influence the extrapolation of the results to clinical reality.

## Conclusions

NaOCl 0.25% was most effective than the other solutions in reduction of CFU count, cell metabolism, hyphae growth, living cells of all *Candida* species and biofilm removal. All solutions have not changed the enzymes productions by *C. albicans* and *C. tropicalis*, but NaOCl caused increased in proteinase production by *C. glabrata*. CT 0.5% was effective in decreasing of CFU count of *C. tropicalis* and cell metabolism of *C. albicans* and *C. glabrata*. RC 10% reduced only the CFU count of *C. glabrata*, but decrease the cellular metabolism of *C. albicans* and *C. glabrata*.

CT0.5% was better than RC10% in the biofilm removal and decrease of living cells of *C. albicans* and *C. tropicalis*, whereas RC 10% was more effective in the biofilm removal of *C. glabrata*. CT 0.5% and RC 10% were similar for the number of *C. glabrata* living cells and hyphal growth of *C. albicans*. In general, the Chloramine T at 0.5% achieved better results than *Ricinus communis* at 10%, favoring the creation of specific products for denture users. Adjustments in the formulations of RC 10% are necessary.
